# Burnout and coping strategies among health system pharmacists in Lebanon: a cross-sectional study

**DOI:** 10.1186/s12913-023-09422-7

**Published:** 2023-05-02

**Authors:** Rosa Abilmona, Hani Dimassi, Rafah Aboulhosn, Nibal Chamoun

**Affiliations:** 1 Lebanese American University Medical Center-Rizk Hospital, Department of Pharmacy, Beirut, Lebanon; 2 Lebanese American University, School of Pharmacy, P.O. Box S-23, Byblos, Lebanon

**Keywords:** Burnout, Hospital, Pharmacists

## Abstract

**Background:**

Burnout in health system pharmacists has been studied in several countries. To date, no data exists on burnout among healthsystem pharmacists in Lebanon. This study aimed to determine the prevalance of burnout, identify factors and describe coping strategies related to burnout among healthsystem pharmacists in Lebanon.

**Methods:**

A cross-sectional study utilizing the Maslach Burnout Inventory- Human Services Survey for Medical Personnel (MBI-HSS (MP))was conducted in Lebanon. A convenience sample of hospital pharmacists in Mount Lebanon and Beirut area filled a paper-based survey in person or via a phone interview. Burnout was defined as having an emotional exhaustion score ≥ 27 and/or depersonalization score ≥ 10. To identify factors associated with burnout, the survey also contained questions on socio-demographic characteristics, professional status, hospital characteristics, professional stressors and professional satisfaction. Participants were also asked about their coping strategies. To adjust for potential confounding, a multivariable logistic regression was used to estimate the adjusted odds ratios of factors and coping strategies associated with burnout. The authors also evaluated burnout according to the broader definition, emotional exhaustion score ≥ 27 or depersonalization score ≥ 10 or low personal accomplishment ≤ 33.

**Results:**

Of the 153 health system pharmacists who were contacted, 115 filled the survey (response rate of 75.1%). The overall burnout prevalence reported was n = 50 (43.5%) and was largely driven by high emotional exhaustion n = 41(36.9%). Following multivariate logistic regression, seven factors were associated with increased burnout: older age, B.S. in Pharmacy degree, involvement in student training, no involvement in procurement, divided attention at work, overall dissatisfaction with career, dissatisfaction to neutrality with balance between professional and personal life. Low personal accomplishment was noted in n = 55 (49.5%). The main coping strategies identified were holidays, leisure, hobbies, sports activities, and relaxation. There was no association between the coping strategies used and burnout. The prevalence of burnout according to the broader definition was n = 77 (67%). The factors associated with the broader definition of burnout were older age, overall dissatisfaction with career and dissatisfaction with work life balance.

**Conclusion:**

Approximately n = 50(43.5%)of health system pharmacists in Lebanon may be at risk for burnout. If using broader definitions incorporating all three subscales of the (MBI-HSS (MP)), the prevalence of burnout was n = 77(67%). This study highlights the need to advocate for pratice reforms to improve the low personal acoomplishment and recommends strategies to mitigate burnout. Further research to assess the current prevalence of burnout and evaluate effective interventions in alleviating burnout amongst health system pharmacists is needed.

**Supplementary Information:**

The online version contains supplementary material available at 10.1186/s12913-023-09422-7.

## Background

Burnout is defined as a psychological syndrome that develops in response to chronic work-related stress. The three key elements of burnout include: emotional exhaustion, depersonalization, and reduced personal accomplishment. Burnout is considered an occupational hazard for various people-oriented professions like human services, education, and health care [1, 2]. Risk factors that contribute to burnout include an increase in workload, lack of control in the workplace, insufficient reward, lack of social support system, absence of fairness, incongruity between personal values of individual and values of the organization, and misalignment between personality and job [3].

The importance of well-being amongst healthcare providers has recently been heavily focused on with a call to implement mitigation strategies to prevent the risk of burnout [4, 5]. Burnout not only affects the physical wellbeing, but has also been correlated with patient safety, namely, self-reported errors, higher staff turnover, and increased mortality ratios in hospitalized patients [6–8].

Recent systematic reviews of burnout in pharmacists, have reported between 10 and 61% of burnout during the pre-covid and covid times [5, 9]. The more recent worldwide data reported 51% burnout amongst 11,306 pharmacists across eight countries. Longer working hours, less professional experience, heavy workload and poor work-life balance were associated with burnout [5]. Other demographic characteristics such as early in career, unmarried or competitive personalities have also been associated with burnout [10]. Moreover, lack of protected administration and teaching time, work conflicts, unaccounted for duties not directly related to primary job functions, and lack of recognition for personal efforts have also been associated with burnout [11, 12]. Interestingly, pharmacists who are truly dedicated to patient care may also be at risk of burnout given the lack of boundaries between work and life [13].

Not only does burnout affect patient safety, but it also has professional, organizational, and personal consequences [14]. On a personal level, some of the noted consequences are anxiety and less self-care [14]. On a professional level, although the impact of burnout in pharmacists has not been as studied as in other healthcare professionals, burnout poses major challenges to healthcare systems, as it has been recognized as a major predictor of turnover among physicians, interrupting continuity-of-care relationships and contributing to the high cost associated with recruiting new clinicians and staff [15, 16].

The implementation of solutions to mitigate burnout have been suggested by a wealth of literature. This includes but is not limited to implementing changes in the workplace to allow pharmacists to work at the top of their license, advocating for a better work-life balance, being appreciative of employees and supporting passionate pharmacists as they advance the practice model [14]. Data regarding the prevalence of burnout in Lebanon has been assessed in physicians, nurses, and medical students as well as community pharmacists, however, there is no baseline data assessing burnout in health-system pharmacists [17–21]. The aim of this study was to determine the prevalence of burnout amongst health system pharmacists in Lebanon, and to identify factors and coping strategies associated with burnout.

## Methods

A cross-sectional study was conducted over an 11 month period, from October 2019 to September 2020, with the majority of participant recruitment during the period between October 2019 to February 2020. Participant recruitment was halted between March till July and resumed in August 2020 given disruptions due to COVID-19. A list of all health system pharmacists working at hospitals in Mount Lebanon and Beirut was obtained from the Order of Pharmacists of Lebanon (OPL), the official association of pharmacists in Lebanon. Pharmacists were approached to complete a 25-minute paper-based survey either in person or via a phone interview. Participation was voluntary and no identifiable variables (such as name or phone numbers) were collected to ensure privacy. The return of the questionnaires constituted informed consent. The protocol was reviewed and approved by the Investigational Review Board (IRB) at the Lebanese American University with study approval code LAU.SOP.RA1.30/Sep/2019.

Only English-speaking health system pharmacists, defined as either hospital pharmacists, clinical pharmacists, or pharmacy residents with at least one year of work experience at hospitals in Mount Lebanon and Beirut were included. The English version was used as the majority of pharmacy schools in Lebanon use English as the medical language [22]. Also most health care professionals in Lebanon are either trilingual or bilingual in Arabic, English and French [23]. Pharmacy technicians and pharmacy students were excluded.

The survey was designed based on the Maslach Burnout Inventory- Human Services Survey for Medical Personnel (MBI-HSS (MP)) and other published studies [12, 13, 24, 25]. The survey consisted of 7 sections: socio-demographic characteristics (age, sex, tobacco, and alcohol habits), professional status (including professional occupation, years of experience as a pharmacist, highest pharmacy degree, number of hours worked per week), hospital characteristics (including primary practice setting, size of the institution, mean daily patient workload), professional stressors (including too many working hours, inadequate research or administrative time, low salary, too many non-clinical duties), professional satisfaction (including overall career, balance between professional and personal life, intellectual challenge of work), MBI-HSS (MP) survey, and coping strategies (including medical consultation, sports activities, hobbies, holidays). Refer to Additional File 1 for the complete survey sections. The investigators used the MBI-HSS (MP) survey, a validated 22-item questionnaire considered to be the gold standard tool for assessing burnout in medical personnel. The questionnaire is answered on a 7 point Likert scale from: never (0) to every day (6). Based on the responses to all questions, point totals were recorded and scores for each of the three domains (emotional exhaustion, depersonalization, and personal accomplishment) were generated [2].

The primary outcome was the prevalence of burnout. Burnout was defined as having an emotional exhaustion score ≥ 27 and/or depersonalization score ≥ 10 on the MBI-HSS (MP) survey [2]. Secondary outcomes included the identification of potential risk factors and coping strategies associated with burnout in Lebanon. Other secondary outcomes included reporting the prevalence of burnout accounting for any of the three subscales on the MBI-HSS (MP) survey [2].

Based on previous studies in similar populations, which reported an overall burnout of 61.2% and 53.2%, a burnout percentage of 50% was considered [11, 12]. A sample size of 165 health system pharmacists was needed for a 95% confidence interval with 7.5% precision. To account for possible refusal to participate, missing information, and data loss, and to avoid over sampling from one large academic medical center, a total of 200 health system pharmacists were contacted to participate in the study. Data were coded and entered into SPSS for analysis. Burnout scores for each of the three domains were generated by the summation of points for the respective questions. Differences in means in burnout scale were tested using the independent t test or the ANOVA F test, while differences in proportions were tested using the Pearson’s Chi-square. All analyses were conducted at the 0.05 significance level. Bivariate analysis was run for each factor (any question that was not part of the MBI) as a potential predictor variable with burnout and no burnout as the dependent variables. Potential predictors with a p-value less than 0.2 in the bivariate analysis were included in a preliminary multiple logistic regression model. The multivariate logistic regression model was repeated using the broad definition of burnout (any of the 3 subscales) part of the sensitivity analysis.

## Results

A total of 200 out of the 220 health system pharmacists working in Beirut and Mount Lebanon area were contacted to participate in the survey. Forty- seven health system pharmacists were not contacted for reasons like not being able to reach or change of their work. As such, a total of 153 pharmacists were successfully contacted, of which, 115 filled the survey (response rate of 75.1%).

Out of the 115 respondents, most were younger than 45 years (n = 94, 81.7%), females (n = 94, 81.7%), with no children (n = 77, 67.0%), and had some kind of social support (n = 82, 71.3%). They were mainly hospital pharmacists (n = 72, 62.6%), worked full-time (n = 114, 99.1%), had a Pharm.D. degree (n = 66, 58.4%), and no certification through board of pharmacy specialties (n = 96, 85.7%). Most pharmacists had practiced for 5 years or less (n = 55, 48.7%) and had practiced in the same hospital for no more than 2 years (n = 43, 37.4%). The majority reported working more than 40 h per week (n = 71, 62.8%). A detailed summary of personal and practice characteristics for all respondents is described in Table 1. Among the proposed coping strategies, taking holidays or time off, leisure, hobbies, sports activities, and relaxation strategies were the most commonly reported (in more than 50% of the respondents) (Table 2).

**Table 1:** Characteristics of Respondents Overall and Based on Burnout

	No. (%) Respondents			
Characteristics	Burnout *(n =* 50)	No Burnout (*n* = 65)	Overall (*n* = 115)	p-value
Age group, year				0.949
< 45	41 (82.0%)	53 (81.5%)	94 (81.7%)	
≥ 45	9 (18.0%)	12 (18.5%)	21 (18.3%)	
Gender				0.162
Female	38 (76.0%)	56 (86.2%)	94 (81.7%)	
Male	12 (24.0%)	9 (13.8%)	21 (18.3%)	
Marital status				0.947
Married	22 (44.0%)	29 (44.6%)	51 (44.3%)	
Single or divorced	28 (56.0%)	36 (55.4%)	64 (55.7%)	
Children	13 (26.0%)	25 (38.5%)	38 (33.0%)	0.159
Smoking	9 (18.0%)	9 (13.8%)	18 (15.7%)	0.543
Alcohol				0.517
None	31 (62.0%)	33 (51.5%)	64 (56.1%)	
Regular (once per week or more)	6 (12.0%)	11 (17.2%)	17 (14.9%)	
Non-regular	13 (26.0%)	20 (31.3%)	33 (28.9%)	
Primary position				0.612
Hospital pharmacist	30 (60.0%)	42 (64.6%)	72 (62.6%)	
Clinical pharmacist	20 (40.0%)	23 (35.4%)	43 (37.4%)	
Employment type				0.252
Full time	49 (98.0%)	65 (100%)	114 (99.1%)	
Part time	1 (2.0%)	0 (0%)	1 (0.9%)	
Years practiced as a licensed pharmacist				0.420
≤ 5	27 (55.1%)	28 (43.8%)	55 (48.7%)	
6–20	15 (30.6%)	27 (42.2%)	42 (37.2%)	
> 20	7 (14.3%)	9 (14.1%)	16 (14.2%	
Years worked in same hospital				0.454
≤ 2	22 (44.0%)	21 (32.3%)	43 (37.4%)	
2–5	10 (20.0%)	16 (24.6%)	26 (22.6%)	
6–15	11 (22.0%)	21 (32.3%)	32 (27.8%)	
≥ 16	7 (14.0%)	7 (10.8%)	14 (12.2%)	
Times changed job				0.980
0	20 (40.0%)	25 (39.1%)	45 (39.5%)	
1	13 (26.0%)	15 (23.4%)	28 (24.6%)	
2	9 (18.0%)	13 (20.3%)	22 (19.3%)	
≥ 3	8 (16.0%)	11 (17.2%)	19 (16.7%)	
Highest pharmacy degree				0.036
B.S.	19 (38.8%)	15 (23.4%)	34 (30.1%)	
Pharm.D. +/- PGY-1	22 (44.9%)	44 (68.8%)	66 (58.4%)	
Other^a^	8 (16.3%)	5 (7.8%)	13 (11.5%)	
BPS certification				0.586
Yes	6 (12.2%)	10 (15.9%)	16 (14.3%)	
No	43 (87.8%)	53 (84.1%)	96 (85.7%)	
School of pharmacy				0.252
American	32 (68.1%)	34 (53.1%)	66 (59.5%)	
Non-American	14 (29.8%)	29 (45.3%)	43 (38.7%)	
Abroad	1 (2.1%)	1 (1.6%)	2 (1.8%)	
Hours worked per week				0.385
≤ 40	16 (32.7%)	26 (40.6%)	42 (37.2%)	
> 40	33 (67.3%)	38 (59.4%)	71 (62.8%)	
Average weekends worked per year				0.878
0	8 (16.7%)	11 (17.5%)	19 (17.1%)	
1–23	19 (39.6%)	22 (34.9%)	41 (36.9%)	
≥ 24	21 (43.8%)	30 (47.6%)	51 (45.9%)	
Average days on call per month				0.332
0	17 (36.2%)	33 (52.4%)	50 (45.5%)	
1–2	11 (23.4%)	8 (12.7%)	19 (17.3%)	
3–5	6 (12.8%)	4 (6.3%)	10 (9.1%)	
6–10	4 (8.5%)	6 (9.5%)	10 (9.1%)	
> 10	9 (19.1%)	12 (19.0%)	21 (19.1%)	
Hours worked weekly from home				0.632
0	19 (40.4%)	31 (48.4%)	50 (45.0%)	
1–5	21 (44.7%)	23 (35.9%)	44 (39.6%)	
> 6	7 (14.9%)	10 (15.6%)	17 (15.3%)	
Social support				0.356
None	16 (32.0%)	17 (26.2%)	33 (28.7%)	
Family	28 (56.0%)	44 (67.7%)	72 (62.6%)	
Non-family	6 (12.0%)	4 (6.2%)	10 (8.7%)	
Practice setting				
Teaching hospital	35 (70.0%)	40 (62.5%)	75 (65.8%)	0.402
Private hospital	43 (86.0%)	54 (84.4%)	97 (85.1%)	0.809
Location				0.536
Beirut	31 (62.0%)	36 (56.3%)	67 (58.8%)	
Mount Lebanon	19 (38.0%)	28 (43.8%)	47 (41.2%)	
Number of beds				0.562
< 50	2 (4.1%)	6 (9.2%)	8 (7.0%)	
50–99	8 (16.3%)	12 (18.5%)	20 (17.5%)	
100–199	21 (42.9%)	21 (32.3%)	42 (36.8%)	
≥ 200	18 (36.7%)	26 (40.0%)	44 (38.6%)	
Mean daily patient workload				0.712
1–30	14 (31.8%)	19 (29.2%)	33 (30.3%)	
31–60	19 (43.2%)	25 (38.5%)	44 (40.4%)	
≥ 61	11 (25.0%)	21 (32.3%)	32 (29.4%)	
Mean daily number of medication orders verified				0.689
None	1 (2.3%)	2 (3.4%)	3 (2.9%)	
≤ 50	17 (38.6%)	16 (27.6%)	33 (32.4%)	
51 − 100	11 (25.0%)	16 (27.6%)	27 (26.5%)	
> 100	15 (34.1%)	24 (41.4%)	39 (38.2%)	
Daily duties				
Order verification	47 (94.0%)	62 (96.9%)	109 (95.6%)	0.652
Pharmacy committee involvement	30 (60.0%)	39 (62.9%)	69 (61.6%)	0.753
Medical staff committee involvement	25 (50.0%)	34 (53.1%)	59 (51.8%)	0.740
Residency precepting	11 (22.0%)	14 (22.2%)	25 (22.1%)	0.977
Student pharmacist precepting	28 (56.0%)	35 (55.6%)	63 (55.8%)	0.962
Student training	36 (72.0%)	34 (54.0%)	70 (61.9%)	0.050
Directing/coordinating a residency program	5 (10.0%)	4 (6.3%)	9 (8.0%)	0.477
Research and publication	19 (38.0%)	25 (39.7%)	44 (38.9%)	0.855
Medication reconciliation	40 (80.0%)	55 (87.3%)	95 (84.1%)	0.292
Formal pharmacist consultation	24 (49.0%)	38 (60.3%)	62 (55.4%)	0.231
Nutrition support consultation	8 (6.3%)	15 (23.8%)	23 (20.5%)	0.331
Didactic lecturing	26 (55.3%)	30 (48.4%)	56 (51.4%)	0.473
Procurement of medications	20 (40.0%)	35 (55.6%)	55 (48.7%)	0.100
Medication dispensing	43 (86.0%)	55 (87.3%)	98 (86.7%)	0.839
Sterile compounding	25 (50.0%)	24 (38.1%)	49 (43.4%)	0.205
Leadership role in formulary management	27 (54.0%)	36 (57.1%)	63 (55.8%)	0.738
Antibiotic stewardship involvement	38 (77.6%)	41 (66.1%)	79 (71.2%)	0.187
Interdisciplinary patient rounds	22 (44.0%)	29 (46.0%)	51 (45.1%)	0.829
Patient counseling	23 (46.0%)	27 (42.9%)	50 (44.2%)	0.738

Abbreviations: BPS, board of pharmacy specialties; PGY-1, postgraduate year 1.

^a^Other includes masters in clinical pharmacy (mainly), masters in hospital pharmacy management, or a PhD

**Table 2:** Coping Strategies against Burnout

	No. (%) Respondents			
	Burnout *(n =* 50)	No Burnout *(n =* 65)	Overall *(n =* 115)	p-value
Holidays or time off	43 (86.0%)	52 (83.9%)	95 (82.6%)	0.755
Leisure	41 (83.7%)	49 (80.3%)	90 (78.3%)	0.651
Hobbies	38 (76.0%)	50 (82.0%)	88 (76.5%)	0.440
Sports activities	30 (60.0%)	34 (54.8%)	64 (55.7%)	0.583
Relaxation strategies	30 (60.0%)	33 (53.2%)	63 (54.8%)	0.472
Nutritional strategies	24 (48.0%)	24 (39.3%)	48 (41.7%)	0.360
Medical consultation	7 (14.0%)	6 (9.7%)	13 (11.3%)	0.478
Medication use	6 (12.0%)	7 (11.3%)	13 (11.3%)	0.907
Psychotherapy	2 (4.0%)	3 (4.8%)	5 (4.3%)	0.831

The overall burnout prevalence among health system pharmacists was (n = 50, 43.5%)(Table 3) and was largely driven by high emotional exhaustion (n = 41, 36.9%). Reduced personal accomplishment was reported in (n = 55,49.5%) of pharmacists. When burnout was evaluated based on the definition of having any of the three criteria, accounting for low personal accomplishment, the prevalence results were (n = 77,66.9%) of pharmacists at risk of burnout. A breakdown of each MBI subscale with the corresponding number of pharmacists who responded as having a low, moderate, or high score is represented in Table 3.

**Table 3:** Assessment of Burnout among Health system Pharmacists (*n* = 115)

Burnout Indices	Median score		No. (%)
Emotional exhaustion		25	
Low score (≤ 18)			33 (29.7%)
Moderate score			37 (33.3%)
High score (≥ 27)			41 (36.9%)
Depersonalization		4	
Low score (≤ 5)			64 (57.7%)
Moderate score			25 (22.5%)
High score (≥ 10)			22 (19.8%)
Personal accomplishment		34	
Low score (≤ 33)			55 (49.5%)
Moderate score			27 (24.3%)
High score (≥ 40)			29 (26.1%)
Burned out^a^		_	50 (43.5%)

^a^Burnout was defined as an emotional exhaustion score ≥ 27 and/or depersonalization score ≥ 10.

**Fig. 1:**
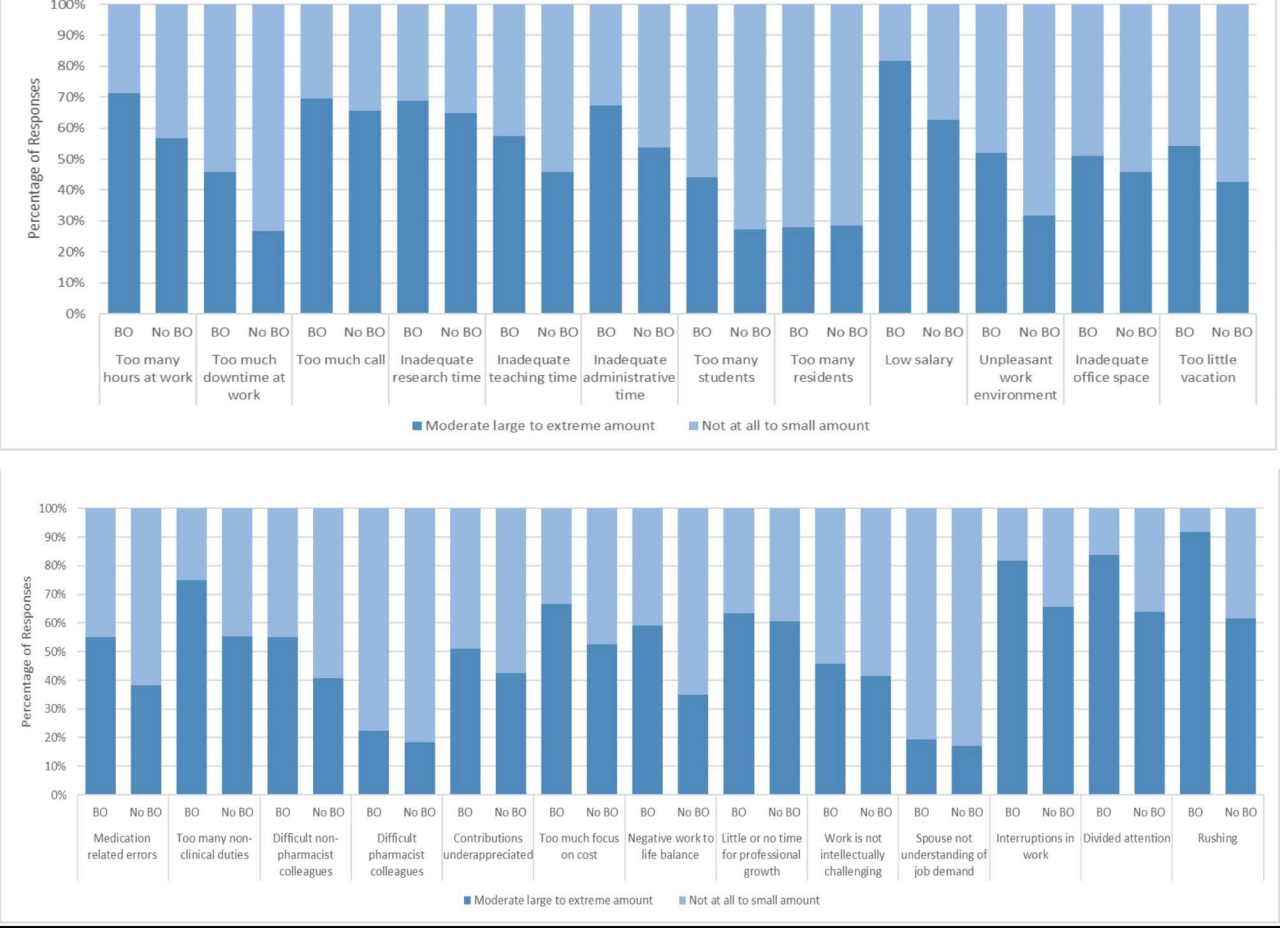
Comparison of the professional stressors responses between the group with burnout (BO) and the group with no BO Burnout (BO), no Burnout (No BO)

In the bivariate analysis, there were no differences observed in the burnout analysis related to demographic characteristics or hospital characteristics. A smaller percentage of those who were burned out had a PharmD as their highest pharmacy degree as compared to those who were not burned out (n = 22, 44.9% vs. n = 44, 68.8%; P = 0.036). Pharmacists who were burned out were also more likely to be involved in student training (n = 36, 72.0% vs. n = 34, 54.0%; P = 0.050). The results of the bivariate analysis on characteristics of respondents based on burnout are presented in Table 1. As for the bivariate analysis on the relation of professional stressors to burnout, too much downtime at work (P = 0.047), low salary (P = 0.030), unpleasant work environment (0.032), too many non-clinical duties (P = 0.034), a negative work to life balance (P = 0.012), divided attention at work (P = 0.021), and rushing (P < 0.001) were found to be associated with burnout. A detailed breakdown of responses for the professional stressors against burnout is shown in Fig. 1. There was no association between the coping strategies and burnout as evident in Table 2.

Seven factors of significance were found in the multivariate regression analysis to increase the risk of burnout (Table 4). Age (OR: 7.41, P = 0.020, 95% CI = 1.37–39.98), having a B.S. Pharmacy degree compared to a Pharm.D. degree (OR: 7.84, P = 0.005, 95% CI = 1.89–32.47), involvement in student training (OR: 5.09, P = 0.015, 95% CI = 1.36–19.04), no involvement in procurement (OR: 11.77, P = 0.001, 95% CI = 2.62–52.93), having divided attention at work (OR: 4.01, P = 0.041, 95% CI = 1.06–15.21), overall dissatisfaction with career (OR: 48.34, P = 0.001, 95% CI = 4.86-481.35), and dissatisfaction to neutrality with balance between professional and personal life (OR: 9.36, P < 0.001, 95% CI = 2.82–31.07) were associated with a higher risk for burnout. When low personal accomplishment < 33 was considered as a part of the burnout definition, the following factors of significance remained in the model as a risk of burnout, age (OR:6.24, P = 0.015, 95% CI = 1.42–27.42), overall dissatisfaction with career (OR: 10.18, P = 0.045, 95% CI = 1.06–98.13), and dissatisfaction to neutrality with balance between professional and personal life (OR: 4.86, P < 0.006, 95% CI = 1.58–14.90) (Table 5).

**Table 4:** Multivariate Logistic Regression Analysis for Pharmacists (*n* = 115)

	Coefficient	Standard error	Odds Ratio (OR)	95% confidence interval	p-value
Age (ref: < 45)					
≥ 45	2.003	0.86	7.41	1.37–39.98	0.020
Education (ref: Pharm.D.)					0.005
B.S.	2.059	0.725	7.84	1.89–32.47	0.005
Other^a^	2.418	0.914	11.23	1.87–67.39	0.008
Student training (ref: no)	1.628	0.673	5.09	1.36–19.04	0.015
No involvement in procurement (ref: yes)	2.465	0.767	11.77	2.62–52.93	0.001
Divided attention at work (ref: no)	1.388	0.681	4.01	1.06–15.21	0.041
Overall dissatisfaction with career (ref: neutral-satisfied)	3.878	1.173	48.34	4.86-481.35	0.001
Dissatisfied-neutral in balance between professional and personal life (ref: satisfied)	2.237	0.612	9.36	2.82–31.07	< 0.001

^a^Other includes masters in clinical pharmacy (mainly), masters in hospital pharmacy management, or a PhD

**Table 5:** Multivariate Logistic Regression Analysis for Pharmacists when burnout defined as emotional exhaustion score ≥ 27 and/or depersonalization score ≥ 10 and/or personal accomplishment ≤ 33 on the MBI-HSS (MP) survey. (*n* = 115)

	Coefficient	Standard error	Odds Ratio (OR)	95% confidence interval	p-value
Age (ref: < 45)					
≥ 45	1.831	0.755	6.241	1.42–27.424	0.015
Education (ref: Pharm.D.)					
B.S.	0.651	0.587	1.917	0.606–6.062	0.268
Other^a^	0.901	0.791	2.462	0.522–11.614	0.255
Student training (ref: no)	0.888	0.537	2.431	0.849–6.961	0.098
No involvement in procurement (ref: yes)	0.767	0.591	2.153	0.676–6.858	0.195
Divided attention at work (ref: no)	0.12	0.545	1.128	0.387–3.286	0.825
Overall dissatisfaction with career (ref: neutral-satisfied)	2.321	1.156	10.183	1.057–98.135	0.045
Dissatisfied-neutral in balance between professional and personal life (ref: satisfied)	1.58	0.572	4.855	1.582–14.895	0.006

^a^Other includes masters in clinical pharmacy (mainly), masters in hospital pharmacy management, or a PhD

## Discussion

The study utilized the validated MBI-HSS designed for medical personnel to assess the prevalence of burnout among health system pharmacists in Lebanon. The results of the study reveal that around 45–67% of health system pharmacists working in Mount Lebanon and Beirut are at risk of experiencing burnout, depending on the definition of burnout used. Although the percentage of burnout, in accordance with the primary outcome, was lower than that seen among pharmacists in previously published articles with similar definitions of burnout, the individual scores of the emotional exhaustion and depersonalization were similar to what was reported in health-system pharmacists [11, 12, 26]. Reduced personal accomplishment was higher than that found in health-system and hospital pharmacists but similar to what was reported in clinical pharmacists [11, 12, 26]. This finding is probably reflective of the impact that the economic collapse has had on mental health as well as the dissatisfaction with career opportunities for health system pharmacists in Lebanon [27–29]. It has been suggested that reduced personal accomplishment is likely to occur when pharmacists cannot focus on their clinical duties and have expectations to non-clinical duties such as working on tasks that can be performed by other members or student training without dedicated time. When pharmacists engage in tasks that can be achieved by others, this also adds to the frustration of not functioning at the top of pharmacists licensure, adding to reduced personal accomplishment [30]. This finding represents an opportunity to advocate for registered pharmacy technician programs and recognized roles to alleviate non-clinical tasks for pharmacists [30, 31]. Pharmacists’ divided attention due to external mental task demands such as interruptions, divided attention, rushing have also been associated with increased burnout, which was also consistent with our findings [32].

Similar to findings in other health care professionals in Lebanon, older age was also associated with an increased risk of burnout. Studies of nurses in Lebanon showed that older age was significantly associated with reduced personal accomplishment [33]. Older age remained associated with burnout even after using the expanded definition, incorporating reduced personal accomplishment. Contrary to our findings, age was protective against burnout amongst clinical pharmacists in the US [11]. This difference is probably due to better career satisfaction and development opportunities available in the US and Europe [27, 34]. More than half of our study represented early-career pharmacists. Recent global literature has shown that pharmacists in the East Mediterranean had higher career expectations, felt significantly less satisfied with their job and had less development and training opportunities as compared with other countries [34]. Given that career dissatisfaction was associated with a risk of burnout in our study and based on global and national findings, imminent changes are needed to provide pharmacists with opportunities for education and training. Practice development nationwide is needed to provide opportunities for advanced practice and professional growth [27, 34]. Moreover, not only should more educational opportunities become available, but employers should also account for professional development time since this has also been noted as a challenge [35].

Moreover, when pharmacists are involved in student training the effects on burnout have been conflicting [26]. Having students incorporated into the practice model to improve the practice setting as pharmacy extenders has been protective, however when the student training time is not accounted for, and perceived as an extra task, this has been negatively associated with burnout [26]. Student training did not remain within the model when low personal accomplishment was accounted for. Perhaps this finding suggests that student training is rewarding to pharmacists in Lebanon.

Congruent to our findings showing the protective role of a Pharm.D. degree on burnout, in a study of recent pharmacy graduates, pharmacists with a Pharm.D. degree were involved less in distributive roles and were thus at lower degree of burnout [36]. Although the clinical role has shown to be protective against the risk of burnout, our study showed that pharmacists involved in procurement were not associated with burnout [26]. An explanation of this finding is that in Lebanon, chief pharmacists are usually involved in procurement, and the lower risk of burnout in this population could be due to other factors like more years of experience or higher salaries. Our survey did not differentiate between a chief pharmacist and a hospital pharmacist and thus we could not identify what was contributing to less burnout in this population. Having post graduate degrees such as masters of doctors of philosophy (PhDs) were also negatively associated with burnout. This is probably due to the lack of career progression and opportunities for growth in health system pharmacy as mentioned above.

The need to balance career and life fulfillment has been well documented in the literature [37–39]. In our study, both dissatisfaction with career and work life balance were associated with an increased risk of burnout. Focusing on extrinsic factors in the workplace such as peer support, job autonomy, institutional culture, wellness training organized in the workplace, and other available resources help pharmacists stay motivated and satisfied [40]. Other strategies proposed to improve work life balance recommend planning and addressing both professional and personal fulfillment [39]. Dissatisfaction with work-life balance also remained in the model when low personal accomplishment was accounted for as a part of the MBI assessment.

Although the percentage of burnout in our study was similar to the percentage of burnout documented during the COVID-19 pandemic, the majority of the participants were recruited by February 2020 before the first case of COVID-19 was identified in Lebanon on February 21st 2020 (Public Health Ministry and Information Ministry, 2020), however, the country had been tremendously impacted by the economic collapse and finally the Beirut blast that occurred on August 4 2020 [5, 29].

This study did not find an association between burnout and coping strategies used by health system pharmacists. In a previous study in French community pharmacists, those with burnout were less involved in leisure activities, sport activities, psychotherapy, and holidays or time off [24].

Based on the findings of this study, there is an urgent need to address pharmacist burnout in Lebanon. The 2019 National Academy of Medicine (NAM) consensus study recommended individual-focused interventions to reduce burnout, combined with effective organizational or system-level interventions. Individual interventions target clinicians’ behaviors and coping strategies, stress and burnout reactions, and resiliency. Mindfulness, stress management, and small-group discussion interventions were the individual-focused approaches that demonstrated the most promise at reducing burnout symptoms. Work-system factors that may improve clinician well-being include enhancing meaning and purpose in work, promoting an organizational culture that supports interprofessional teamwork, collaboration, communication, and professionalism, aligning incentives, compensation, and reward systems for clinicians with organizational and professional values, and providing access to resources, such as coaching and adequate time for professional and personal development [41]. The evidence for system interventions that significantly impact clinician burnout is limited and warrants rigorous research to evaluate the impact of such strategies on burnout [41] Additional research in Lebanon is needed to re-evaluate the prevalence of burnout today. Based on the study findings, we suggest that low personal accomplishment should be incorporated into the definition of burnout when assessing burnout in Lebanon.

To our knowledge, this is the first study to address burnout amongst health system pharmacists in Lebanon. A response rate of 75% was obtained in this study, higher than that found in previous trials [11, 12, 26], mainly due to the direct delivery of the survey to participants who were reluctant to fill it over the phone. The direct follow up with the pharmacists secured a better response. Moreover, this study adds to global literature emphasizing the need to allow pharmacists to work at the top of their license.

The study is not without limitations. The total number of pharmacists initially expected was lower than the actual number and this is due to the fact that the list obtained from the OPL was not updated. The target sample size initially determined was not reached, however a power analysis was rerun with the effect size, the sample size was recalculated to be 144 (less than the initial target). Also, although the time frame for data collection included the start of the COVI-19 pandemic, the majority of the patient recruitment was mainly performed during the first 5 months of the study pre-COVID-19. Another limitation could be some of the phrasing of the MBI-HSS (MP), in which some questions are asked in terms of direct patient care interactions. Given that most of the pharmacy practice models across hospitals in Lebanon do not include direct patient care, these questions may have been answered inaccurately. Moreover, the survey did not assess pharmacists’ awareness of burnout resources. Such data would have provided insight regarding the culture/ work environment and professional development provided at their respective institutions [12, 26].

## Conclusion

At the time of conducting this study, the prevalence of burnout amongst health system pharmacists was 43.5–67%in Lebanon. The authors suspect that currently the prevalence of burnout is even higher given that clinician burnout is a complex multifactorial problem. After the conclusion of this study, Lebanon, and the healthcare sector experienced many stressors including the COVID-19 pandemic that started at the end of February 2020 and the Beirut blast that happened on August 4th 2020. Although many of these factors are external stressors, this study highlights an important career dissatisfaction amongst health system pharmacists. Significant pharmacy practice reform is needed to motivate pharmacists. Additional research is needed to evaluate the prevalence of burnout amongst health system pharmacists today, and to evaluate the impact of system changes on burnout and important outcomes such as medication errors and turnover.

## Electronic supplementary material

Below is the link to the electronic supplementary material.

Supplementary Material 1

## Data Availability

The datasets generated during the study are included in this published article. The datasets analysed during the current study are not publicly available due to the use of select copyrighted questions but are available from the corresponding author on reasonable request.

## List of Abbreviations

OPL: Order of Pharmacists of Lebanon
IRB: Investigational Review Board
MBI-HSS (MP): Maslach Burnout Inventory- Human Services Survey for Medical Personnel
NAM: National Academy of Medicine
