# Supplementary material for: Burnout and coping strategies among health system pharmacists in Lebanon: a cross-sectional study
Source: BMC Health Serv Res. 2023 May 2;23:424. doi: 10.1186/s12913-023-09422-7 (PMC10152435; doi:10.1186/s12913-023-09422-7)
Supplement: Supplementary file 1 — Supplementary Material 1 [file 12913_2023_9422_MOESM1_ESM.pdf]

Appendix

Section 1: Demographic Characteristics

Please complete the following section regarding your demographic data

1) What is your age (years)?

☐ 18-34

☐ 35-44

☐ 45-54

☐ 55-64

☐ 65-70

2) What is your gender?

☐ Male

☐ Female

3) What is your marital status?

☐ Married

☐ Single

☐ Divorced

☐ Widowed

4) Do you have children?

☐ Yes

☐ No

5) Do you smoke?

☐ No

☐ Yes

  - ☐ <1 cigarette pack/day
  - ☐ 1-2 cigarette packs/day
  - ☐ >2 cigarette packs/day

6) Do you drink alcohol?

☐ No

☐ Yes

- ☐ Every day
- ☐ 3- 5 times/ week
- ☐ Once a week
- ☐ Only on weekends
- ☐ On special occasions

Section 2: Professional Status

7) What is your professional occupation?

☐ Hospital pharmacist

☐ Clinical pharmacist

☐ Pharmacy Resident

8) Please select the term that best represents how you are employed in your current position

☐ Full Time

☐ Part Time

9) How many years of experience do you have as a licensed pharmacist?

____

10) For how long have you been working in the same hospital?

____

11) How many times have you changed your job?

____

12) What is your highest pharmacy degree?

☐ BS Pharmacy

☐ PharmD

- PGY-1
- PGY-2
- No postgraduate training

☐ Other

13) Which School of Pharmacy did you graduate from?

____

14) Are you BPS certified?

☐ Yes

☐ No

15) On average, how many hours do you work per week?

☐ < 20

☐ 20-40

☐ 41-60

☐ > 60

16) How many average weekends do you work per year?

____

17) How many hours weekly do you work from home?

☐ 0

☐ 1-5

☐ 6-10

☐ 10-20

☐ ≥21

18) How many average days do you work on call per month?

____

19) Are you eligible to receive overtime pay?

☐ Yes

☐ No

20) Have you ever observed or experienced any kind of discrimination at work? (sexual harassment, religious, gender…)?

____

21) Who is your social support?

____

Section 3: Hospital Characteristics

22) What is your primary practice setting?

☐ Teaching Hospital ☐ Nonteaching Hospital

23) Please indicate which setting describes your practice

☐ Private Hospital ☐ Public Hospital

24) Where is the location of the hospital you practice in?

☐ Beirut ☐ Mount Lebanon

25) What is the size of the institution you work in (number of beds)?

☐ < 50

☐ 50-99

☐ 100-199

☐ ≥ 200

26) What is the mean daily patient workload (i.e. how many patients do you manage per day)?

____

27) What is the mean number of medication orders verified?

____

28) Does your current position involve participation in the following activities?

	**Yes**	**No**
Order Verification		
Pharmacy Committee Participation		
Medical Staff Committee Participation		
Resident Precepting		
Student Precepting		
Student Training		

Directing/Coordinating a residency program		
Research / Publication		
Medication Reconciliation		
Formal Pharmacy Consultation (excluding nutrition)		
Nutrition Support Consultation		
Didactic Lecturing		
Procurement, Selection, and Purchasing of medications		
Medication Dispensing		
Sterile Compounding		
Providing Adequate Information for safe medication use		
Strategies and Policies Implementation to Prevent Medication Errors		
Leadership Role in Formulary Management and Update		
Antibiotic Stewardship Involvement		
Pharmacokinetic Consults		
Pharmacotherapy Consults		
Documentation in Medical Records		
Pharmacy Patient Care Rounds		
Interdisciplinary Patient Care Rounds		
Patient Counseling		

Section 4: Professional Stressors

29) To what extent have the following factors affected you in your practice over the last 12-24 months?

	**Not at all**	**Small amount**	**Moderate amount**	**Large amount**	**Extreme amount**	**Not applicable**
Too many hours at work						
Too much downtime at work						

Too much call						
Inadequate research time						
Inadequate administrative time						
Inadequate teaching time						
Too many students						
Too many residents						
Low salary						
Unpleasant work environment						
Inadequate office space						
Too little vacation						
Medication-related errors						
Too many non-clinical responsibilities						

Difficult nonpharmacist colleagues						
Difficult pharmacist colleagues						
Contributions under-appreciated						
Too much focus on cost						
Negative work/life balance						
Little or no time for professional growth						
Work is not intellectually challenging						
Spouse not understanding of job demand						
Interruptions in work						
Rushing						
Divided Attention						

Section 5: Professional Satisfaction

30) In relation to your current position as a hospital pharmacist, how satisfied are you with the factors listed below?

Please answer by:
5: Very satisfied
4: Somewhat satisfied
3: Neutral
2: Somewhat dissatisfied
1: Very dissatisfied

	**1** Very dissatisfied	**2** Somewhat dissatisfied	**3** Neutral	**4** Somewhat satisfied	**5** Very satisfied
Overall career					
Balance between professional and personal life					
Time for professional growth					
Intellectual challenge of work					
My pharmacist colleagues					
My physician and nursing colleagues					
My salary / income					

Section 6: MBI-HSS MP

Section 7: Coping Strategies

32) What strategies do you use to manage your work-related stress?

	**Yes**	**No**
Medical consultation		
Medication use<br>○ Medical prescription<br>○ Self-medication		
Medication Type (if applicable)<br>○ Anxiolytic medication<br>○ Hypnotic medication<br>○ Phytotherapy medication<br>○ Homeopathy medication<br>○ Others: ___________________________		
Leisure		
Sport activities		
Hobbies		
Holidays or time off		
Nutritional strategies		
Relaxation strategies		
Psychotherapy		
